# Application of Green Tea Catechin for Inducing the Osteogenic Differentiation of Human Dedifferentiated Fat Cells *in Vitro*

**DOI:** 10.3390/ijms161226081

**Published:** 2015-11-25

**Authors:** Koji Kaida, Yoshitomo Honda, Yoshiya Hashimoto, Masahiro Tanaka, Shunsuke Baba

**Affiliations:** 1 Department of Oral Implantology, Osaka Dental University, Osaka 573-1121, Japan; kaida-k@cc.osaka-dent.ac.jp (K.K.); baba-s@cc.osaka-dent.ac.jp (S.B.); 2 Institute of Dental Research, Osaka Dental University, Osaka 573-1121, Japan; 3 Department of Biomaterials, Osaka Dental University, Osaka 573-1121, Japan; yoshiya@cc.osaka-dent.ac.jp; 4 Department of Fixed Prosthodontics and Occlusion, Osaka Dental University, Osaka 573-1121, Japan; tanaka-m@cc.osaka-dent.ac.jp

**Keywords:** polyphenol, catechin, EGCG, bone, dedifferentiated fat cell, stem cells, osteoblast

## Abstract

Despite advances in stem cell biology, there are few effective techniques to promote the osteogenic differentiation of human primary dedifferentiated fat (DFAT) cells. We attempted to investigate whether epigallocatechin-3-gallate (EGCG), the main component of green tea catechin, facilitates early osteogenic differentiation and mineralization on DFAT cells *in vitro*. DFAT cells were treated with EGCG (1.25–10 μM) in osteogenic medium (OM) with or without 100 nM dexamethasone (Dex) for 12 days (hereafter two osteogenic media were designated as OM(Dex) and OM). Supplementation of 1.25 μM EGCG to both the media effectively increased the mRNA expression of collagen 1 (*COL1A1*) and runt-related transcription factor 2 (*RUNX2*) and also increased proliferation and mineralization. Compared to OM(Dex) with EGCG, OM with EGCG induced earlier expression for *COL1A1* and *RUNX2* at day 1 and higher mineralization level at day 12. OM(Dex) with 10 μM EGCG remarkably hampered the proliferation of the DFAT cells. These results suggest that OM(without Dex) with EGCG might be a preferable medium to promote proliferation and to induce osteoblast differentiation of DFAT cells. Our findings provide an insight for the combinatory use of EGCG and DFAT cells for bone regeneration and stem cell-based therapy.

## 1. Introduction

In the past decade, stem cell engineering has undergone significant advances. Various stem cells have been extensively investigated for their potential for use in dental [1] and orthopedic surgery [2] to treat conditions such as inflammatory diseases, trauma, and tumors. So far, induced pluripotent stem cells and somatic stem cells, such as mesenchymal stem cells (MSCs) and adipocyte derived stem cell (ADSCs), that exhibit multipotency and proliferative properties, have garnered great interest in bone regenerative medicine.

Dedifferentiated fat (DFAT) cells isolated from adipose tissues are thought to be a promising cell type for use in stem cell-based regenerative therapy [3]. Mature adipocytes begin to dedifferentiate into elongated non-lipid-filled fibroblast-like cells when cultured in specific conditions such as by using ceiling culture technique [4] and hydrogels [5] *in vitro*. The cells differentiate into multiple cell lineages, including osteoblasts [3,6,7,8,9,10,11,12,13,14,15], chondrocytes [3,14,16], adipocytes [3,15,17], myocytes [14,15,18,19,20], and endothelial cells [21]. Expression markers and proliferative capacity of DFAT cells are analogous to those of MSCs and ADSCs, but are more homogenous [3,22]. The expression of embryonic stem cell markers in DFAT cells was also similar to that in ADSCs [23]. DFAT cells have also been known to be able to differentiate into osteoblasts much earlier than MSCs or ADSCs [8,24].

Epigallocatechin-3-gallate (EGCG), which is the most abundant polyphenol in green tea catechin, has attracted intense interest as a health-promoting agent owing to its anti-inflammatory, anticancer, antioxidant, and anti-atherogenic properties [25]. This polyphenol controls multiple signaling pathways, including the extracellular signal-regulated kinase (ERK) [26], c-Jun N-terminal kinase (JNK) [27,28], mitogen-activated protein/ERK kinase 1/2 [27], signal tranducer and activator of transcription 3 (STAT3) [27,29], and phosphoinositide-3kinase/Akt pathways [26,27,30,31], which are responsible for a variety of cellular responses, including proliferation [27,32], differentiation [31,32,33], cytokine production [28,30], and survival [26]. Therefore, the potential for the therapeutic use of EGCG for various diseases such as carcinomas [30], metabolic syndrome [34], oral diseases [35], cardiovascular diseases [36], and bone diseases [37,38,39] has been extensively studied. 

In bones, EGCG is known to modulate bone metabolism [40], bone formation [37,39], and bone resorption [41] even *in vivo*. In *in vitro* studies, EGCG has been known to hinder osteoclastogenesis [41], while it induces osteoblast differentiation in mesenchymal stem cells [32,42,43] and activates bone-like cells [33,44]. However, there is no study regarding the effect of EGCG on osteoblastic differentiation of DFAT cells. Considering the easier availability of fat tissue in comparison with that of the bone marrow, osteogenically differentiated DFAT cells are a potential and attractive cell source for developing bone regeneration therapies and drug discovery.

Therefore, in this study, we investigated whether EGCG promotes the osteoblast differentiation of primary human DFAT cells using two osteogenic media: (1) OM: osteogenic medium without dexamethasone (Dex); (2) OM(Dex): OM with Dex. (The detailed compositions of the two media have been provided in Table 1). Hereafter in this paper, the media formed by supplementing EGCG in OM or OM(Dex) are designated as OM + EGCG(N) or OM(Dex) + EGCG(N), respectively, where N = concentration of EGCG (μM). To determine the detailed mechanisms underlying the osteogenic capability of EGCG in two media, we used inhibitors of four signal transduction pathways: p38-mitogen-activated protein kinase (p38-MAPK), Akt, ERK1/2, and JNK pathways.

**Table 1:** Medium compositions.

Media	Abbreviation	Composition	Dex
Control medium	Control	Basal Dulbecco’s modified eagle medium with 10% FBS and 1% antibiotics	−
Osteogenic medium 1	OM	Control medium with 50 μM AA2P and 10 mM bGP	−
Osteogenic medium 1 with EGCG	OM + EGCG(N)	OM with 1.25–10 μM EGCG	−
Osteogenic medium 2	OM(Dex)	Control medium with 50 μM AA2P, 10 mM bGP and 100 nM Dex	+
Osteogenic medium 2 with EGCG	OM(Dex) + EGCG(N)	OM(Dex) with 1.25–10 μM EGCG	+

N in EGCG(N): concentration of EGCG (μM); EGCG = epigallocatechin-3-gallate; Dex = dexamethasone; AA2P = ascorbic acid 2-phosphate; FBS = fetal bovine serum; bGP = β*-g*lycerophosphate.

## 2. Results

### 2.1. Effect of Epigallocatechin-3-Gallate (EGCG) on the Proliferation of Dedifferentiated Fat (DFAT) Cells

**Figure 1:**
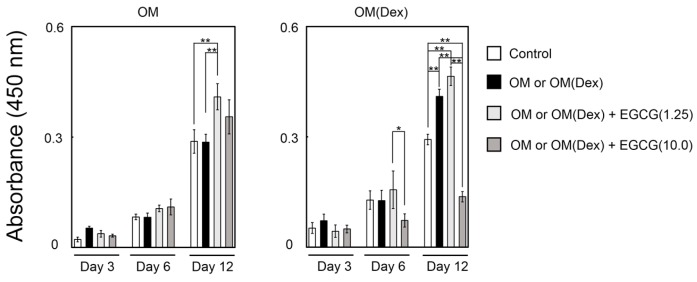
Results of the cell proliferation assay. OM: osteogenic medium without Dex; OM(Dex): OM with 100 nM Dex. Control: control medium. Cells were seeded in condition 1 and were subjected to EGCG in OM or OM(Dex). N in EGCG(N): concentration of EGCG (μM). * *p* < 0.05, ** *p* < 0.01 (Analysis of variance (ANOVA) with a Tukey–Kramer test). The bar graph shows the mean with standard deviation (*n* = 4).

The DFAT cells were cultured in cell seeding condition 1. At day 12, the supplementation of 1.25 μM EGCG most effectively enhanced the proliferation of the cells (Figure 1). Meanwhile, OM(Dex) + EGCG(10.0) resulted in remarkably lower proliferation compared to the control and OM(Dex) treatments. This suggested that excess EGCG in conjunction with Dex is likely to hinder the proliferation of DFAT cells.

### 2.2. mRNA Expression Levels of Osteogenic Markers and Alkaline Phosphatase Assay

Table 2 and Table 3 show the mRNA expression levels of osteogenic markers associated with EGCG-induced osteoblast differentiation of the DFAT cells at days 1 and 6. EGCG(1.25) administration resulted in higher expression of the early osteogenic markers collagen type 1 α 1 (*COL1A1*) and Runt-related transcription factor 2 (*RUNX2*) compared with the expression levels observed with OM and OM(Dex) treatment at day 1. The expression level of OM + EGCG(1.25) was approximately 4-fold higher than that treated with OM(Dex) + EGCG(1.25). At day 6, the expression levels of *RUNX2*, Osterix (*OSX*), and distal-less homeobox 5 (*DLX5)* were upregulated by the treatment with or without EGCG in two different osteogenic media. Earlier expression of Osteocalcin (*OCN*) was observed in the cells treated with EGCG in OM (Table 3).

**Figure 2:**
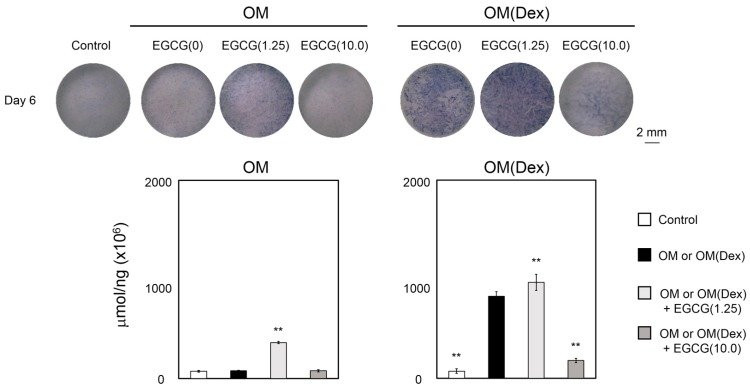
Alkaline Phosphatase (ALP) staining and the corresponding quantitative data for ALP expression of DFAT cells treated with or without EGCG in two different osteogenic media. OM: osteogenic medium without Dex; OM(Dex): OM with 100 nM Dex. Control: control medium. Cells were treated under condition 2. N in EGCG(N): concentration of EGCG (μM). ** *p* < 0.01 (ANOVA with a Tukey–Kramer test) indicates a statistically significant difference against OM or OM(Dex). The bar graph shows the mean with standard deviation (*n* = 4).

Figure 2 shows the alkaline phosphatase (ALP) staining and the corresponding quantitative data for ALP expression of the cells treated with or without EGCG in two different osteogenic media. Strong ALP staining was observed in the cells treated with EGCG(1.25) in both OM and OM(Dex) at day 6. The cells treated with OM(Dex) + EGCG(1.25) showed stronger ALP staining than those cultured with OM + EGCG(1.25).

**Table 2:** mRNA expression levels of osteogenic markers in dedifferentiated fat (DFAT) cells treated with media on day 1.

mRNA	*COL1A1*	*RUNX2*	*OSX*	*DLX5*
Media Methods Units	qPCR Fold (*vs.* Control)	Digital PCR Copy/μL	Digital PCR Copy/μL	Digital PCR Copy/μL
Control	1.08 ± 0.46 ^c,d^	32.99 ± 4.27 ^c^	15.42 ± 1.38 ^d^	22.78 ± 1.85 ^b,c^
OM	3.32 ± 1.20	223.59 ± 15.17	13.33 ± 0.81	32.21 ± 2.55
OM + EGCG(1.25)	31.10 ± 7.93 ^c^	276.72 ± 3.44 ^c^	71.11 ± 20.84 ^c^	26.43 ± 1.05 ^a^
OM + EGCG(10.0)	8.16 ± 5.65	19.33 ± 0.84	21.00 ± 3.39	27.05 ± 1.27 ^a^
OM(Dex)	0.72 ± 0.45	36.11 ± 2.29	9.81 ± 0.01	26.51 ± 0.17
OM(Dex) + EGCG(1.25)	7.07 ± 1.29 ^d^	60.93 ± 4.28 ^d^	12.32 ± 0.07 ^d^	25.58 ± 0.66
OM(Dex) + EGCG(10.0)	1.97 ± 0.72	17.38 ± 0.57 ^d^	18.66 ± 0.77 ^d^	26.16 ± 1.09

OM: osteogenic medium without Dex; OM(Dex): OM with 100 nM Dex. Control: control medium. Cells were treated with Condition 2. N in EGCG(N): concentration of EGCG (μM). Values: mean with standard deviation (*n* = 4). ^a,b^: *p* < 0.05; ^c,d^: *p* < 0.01 (ANOVA with a Tukey–Kramer test). ^a,c^: *vs.* OM; ^b,d^: *vs.* OM(Dex). *COL1A1*: collagen type 1 α 1; *RUNX2*: runt-related transcription factor 2; *OSX*: osterix; *DLX5*: distal-less homeobox 5.

**Table 3:** mRNA expression levels of osteogenic markers in DFAT cells treated with media on day 6.

mRNA	*COL1A1*	*RUNX2*	*OSX*	*DLX5*	*OCN*
Media Units	Fold (*vs.* Control)	Fold (*vs.* Control)	Fold (*vs.* Control)	Fold (*vs.* Control)	Fold (*vs.* Control)
Control	1.02 ± 0.23 ^c,d^	1.17 ± 0.71 ^b,c^	1.01 ± 0.12 ^c,d^	1.02 ± 0.21 ^c,d^	1.16 ± 0.59 ^d^
OM	0.15 ± 0.02	5.00 ± 0.79	20.68 ± 6.58	2.54 ± 0.14	31.74 ± 12.28
OM + EGCG(1.25)	0.27 ± 0.08	4.56 ± 0.72	36.01 ± 3.10 ^c^	3.41 ± 0.15 ^c^	140.46 ± 65.44 ^c^
OM + EGCG(10.0)	0.20 ± 0.03	3.71 ± 1.35	52.57 ± 9.29 ^c^	2.38 ± 0.13	71.83 ± 5.45
OM(Dex)	0.52 ± 0.08	2.79 ± 0.58	16.94 ± 0.71	1.93 ± 0.20	0.43 ± 0.03
OM(Dex) + EGCG(1.25)	0.40 ± 0.04	2.38 ± 0.40	29.62 ± 4.60 ^d^	2.39 ± 0.44	1.03 ± 0.10 ^d^
OM(Dex) + EGCG(10.0)	0.18 ± 0.02 ^d^	3.04 ± 0.89	25.24 ± 0.18 ^d^	2.17 ± 0.04	0.88 ± 0.15 ^d^

OM: osteogenic medium without Dex; OM(Dex): OM with 100 nM Dex. Control: control medium. mRNA expression was evaluated using qPCR. Cells were treated with Condition 2. N in EGCG(N): concentration of EGCG (μM). Values: mean with standard deviation (*n* = 4). ^b^: *p* < 0.05; ^c,d^: *p* < 0.01 (ANOVA with a Tukey–Kramer test). ^c^: *vs.* OM; ^b,d^: *vs.* OM(Dex). *COL1A1*: collagen type 1 α 1; *RUNX2*: runt-related transcription factor 2; *OSX*: osterix; *DLX5*: distal-less homeobox 5; *OCN*: osteocalcin.

### 2.3. Mineralization

**Figure 3:**
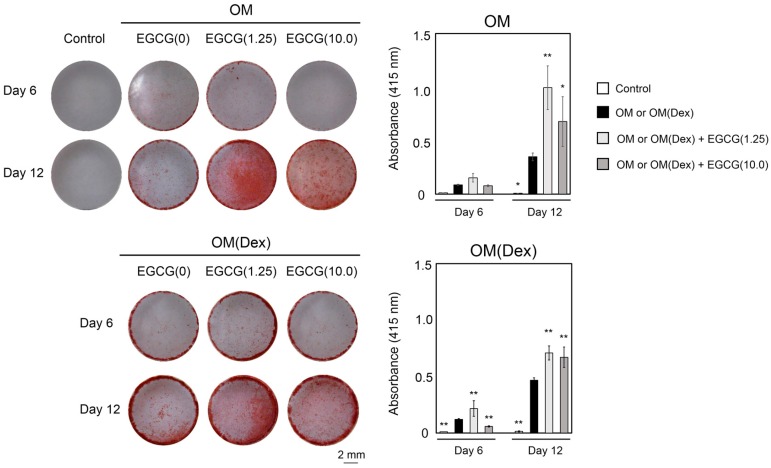
Alizarin red staining of DFAT cells treated with or without EGCG in two different osteogenic media and the corresponding quantitative data. OM: osteogenic medium without Dex; OM(Dex): OM with 100 nM Dex. The cells were treated under condition 2. N in EGCG(N): concentration of EGCG (μM). * *p* < 0.05, ** *p* < 0.01 (ANOVA with a Tukey-Kramer test) indicates a statistically significant difference against OM or OM(Dex). The bar graph shows the mean with standard deviation (*n* = 4).

Mineralization indicated by the intensity of alizarin red staining gradually increased in the cells treated with OM or OM(Dex) with or without EGCG (Figure 3). OM(Dex) without EGCG resulted in higher mineralization than that observed with OM without EGCG. When EGCG was added in two osteogenic media, EGCG(1.25) resulted in significantly higher alizarin red staining compared with that observed with OM or OM(Dex) alone. OM + EGCG(1.25) treatment yielded stronger alizarin red staining than that observed with OM(Dex) + EGCG(1.25), suggesting that supplementation of Dex attenuated the mineralization induced in DFAT cells under the conditions of EGCG stimulation.

### 2.4. Inhibitory Assay to Evaluate EGCG-Induced Osteoblast Differentiation of DFAT Cells

**Figure 4:**
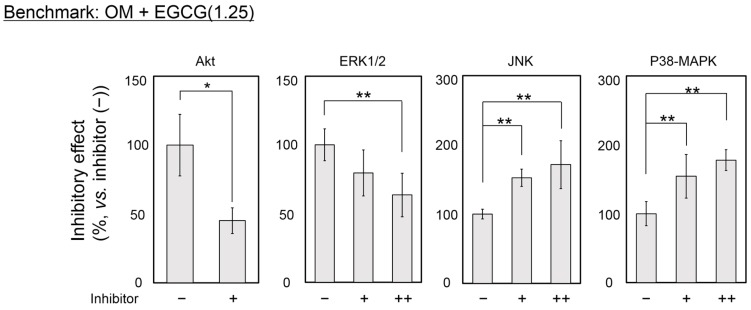

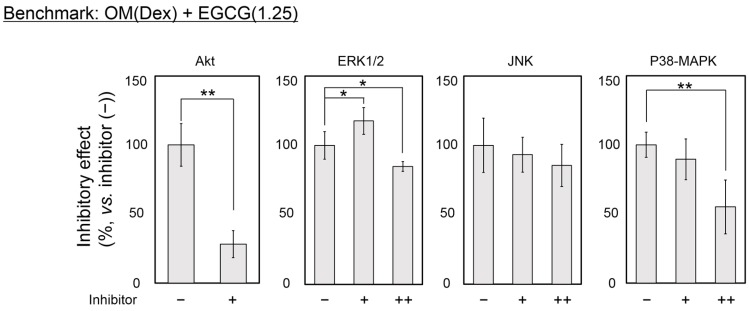
Effect of inhibitors of the extracellular signal-regulated kinase (ERK) 1/2, Akt, c-Jun *N*-terminal kinase (JNK) and p38-mitogen-activated protein kinase (MAPK) signaling pathways on EGCG-induced mineralization. Cells were seeded under condition 2. OM: osteogenic medium without Dex. OM(Dex): OM with 100 nM Dex. The cells were treated with or without inhibitors in OM + EGCG(1.25) or OM(Dex) + EGCG(1.25) for 12 days. N in EGCG(N): concentration of EGCG (μM). The concentrations of the inhibitors were designated as + and ++: 2.5 and 25 µM for the ERK1/2 inhibitor PD98059; 1 and 10 µM for the JNK inhibitor SP600125; and 1 and 10 µM for the p38-MAPK inhibitor SB203580 (the Akt inhibitor API-2 was used at 1 µM, designated as +). The alizarin red staining levels of the samples were normalized against those of the cells treated with OM + EGCG(1.25) or OM(Dex) + EGCG(1.25) without inhibitors. * *p* < 0.05, ** *p* < 0.01 (ANOVA with a Dunnett’s test and Student’s *t*-test). The bar graph shows the mean with standard deviation (*n* = 4).

We further attempted to clarify the mechanisms underlying the osteogenic capability of OM or OM(Dex) with EGCG by using alizarin red staining and inhibitors of four signal transduction pathways: PD98059 for ERK1/2, API-2 for Akt, SB203580 for p38-MAPK, and SP600125 for JNK (Figure 4). Administration of the Akt inhibitor inhibited the mineralization of the cells treated with OM + EGCG(1.25) and OM(Dex) + EGCG(1.25) to a similar level. In contrast, there were obvious differences between the effects of the inhibitors of the ERK1/2, JNK, and p38-MAPK pathways. In particular, the effect of the p38-MAPK inhibitor on the mineralization in the cells treated with OM + EGCG(1.25) was opposite to that observed in the cells treated with OM(Dex) + EGCG(1.25).

## 3. Discussion

In the present study, we showed that the supplementation of EGCG in OM and OM(Dex) resulted in significantly higher proliferation and mineralization, and earlier osteoblast differentiation for DFAT cells than that observed with conventional OM and OM(Dex) alone. OM + EGCG induced earlier osteogenic differentiation and higher mineralization level than that with OM(Dex) + EGCG. Using the inhibitors for four signaling pathways, we found differences in the effect of the ERK1/2, JNK and p38-MAPK pathways on the EGCG-induced mineralization between the cells treated with OM + EGCG and OM(Dex) + EGCG. This result suggested that the supplementation of Dex in OM markedly altered the signaling pathways of DFAT cells, thereby attenuating the osteogenic capability of EGCG.

Although this is the first report that addresses the guiding of cell fate and proliferation of DFAT cells by EGCG, its effects on MSCs and osteoblasts have been previously investigated at the concentration range of 1–100 μM [40]. In previous studies, the concentration superior to induce the mineralization of human MSCs [32] and human osteoblast-like cells [33] was found to be 5 μm, and that for mouse MSCs was found to be 10 μM [42]. Jin *et al.* reported that 10 μM EGCG attenuated cell growth, but that the level of cell growth was still higher than that of cells treated with control media [32]. Consistent with this result, our results showed that EGCG administration induced the mineralization and enhanced the proliferation of DFAT cells at similar concentration ranges as those used in previous studies. However, the strongest induction of mineralization was observed with 1.25 μM EGCG, which is lower than concentration used in previous studies. The proliferation of DFAT cells cultured in OM(Dex) + EGCG(10.0) was remarkably lower than that of cells cultured in the control medium. These results suggest that the DFAT cells responded to EGCG as well as MSCs and osteoblasts, while their susceptibility to the effect of EGCG might be higher than that of MSCs and osteoblasts.

The osteoblast maturation process is known to proceed in the following four steps: lineage commitment, proliferation, maturation, and mineralization [45]. Collagen 1, Runx2, Osterix, and Dlx-5 are mainly expressed in the proliferation or maturation stages [45]. ALP is thought to be a defined marker for osteoblastogenesis [46,47] and is a key regulator that promotes the mineralization of the bone matrix [46,48]. Nevertheless, our results show that the supplementation of Dex into OM + EGCG enhanced the ALP staining level of the cells, but decreased the alizarin red staining level (Figure 2 and Figure 3). This discrepancy might be explained by the possible synergistic effect of EGCG and Dex on the activation of STAT3 signaling. EGCG administration modulates collagen production and proliferation in fibroblasts through STAT3 signaling [27]. In addition, Mikami *et al.* have reported that Dex administration markedly enhances ALP production via activation of STAT3 signaling [47]. Further, Peruzzi *et al.* have reported the possibility that this pathway impairs osteoblast maturation *in vivo* and *in vitro*. *COL1A1* and *Runx2* expression is also known to be increased by STAT3 siRNA treatment [49]. Consistent with the results of these studies, our results show that simultaneous administration of EGCG and Dex in OM remarkably increased ALP staining (Figure 2) and decreased the mRNA expression of *COL1A1* and *RUNX2* as well as mineralization. Although the effect of STAT3 pathways on osteoblastogenesis is still a controversial topic [47,49,50,51], the activation of STAT3 signaling might be partially associated with the attenuated mineralization observed in the DFAT cells treated with a combination of Dex and EGCG in OM in our study.

Cocktails of Dex, AA2P (or ascorbic acid), and bGP have been widely used to supplement media to promote the osteogenic differentiation of multipotent stem cells [52] such as ADSCs [53,54], MSCs [55,56,57], and DFAT cells [3,24]. Supplementation of Dex in the medium is an effective technique to induce the osteoblast differentiation of human MSCs [52,55], wherein Dex acts via the augmentation of the transcripts of the LIM-domain protein with 4.5 LIM domains [52,58]. In agreement with these results, our results showed that stronger mineralization occurred in human DFAT cells treated with OM(Dex) than that observed with OM (Figure 3). However, the results were reversed by the addition of EGCG in two osteogenic media. Using inhibitors of four signal transduction pathways, we found apparent differences in the effects of the ERK, JNK, and p38-MAPK pathways on mineralization between the treatments with OM + EGCG and OM(Dex) + EGCG (Figure 4). The MAPK cascade (ERK, JNK, and P38-MAPK) and Akt signaling modulate processes that determine cell fate, such as proliferation, differentiation, and apoptosis [59,60]. Similar MAPK kinetic profiles often yield opposite cellular fates according to the type of cell and signal intensity. Thus, the network of these pathways has been vigorously investigated [59]. Additionally, it is recognized that the crosstalk between signaling pathways regulates epigenetic mechanisms [61]. Unfortunately, we could not determine the detailed mechanism underlying the osteogenic capability of EGCG in DFAT cells. The optimal harmony between the signaling pathways and epigenetic mechanisms might be associated with the osteogenic capability of OM + EGCG.

A combination of stem cells, scaffold, and osteoinductive reagents is considered as a prospective approach for bone disease repair [62]. Compared with the wide use of growth factors in bone regeneration therapy [62], the application of polyphenols is still limited despite its various advantages such as cost-effectiveness. More recently, we reported that EGCG-conjugated gelatin composites strategically and locally released polyphenol in a critical-sized defect in mouse calvaria, thereby inducing bone formation at a low dose of EGCG [39]. Previous studies have demonstrated the effectiveness of the use of DFAT cells in bone therapies [12,63]. In view of the high accessibility of adipose tissues, preparing a sufficient quantity of DFAT cells would be more feasible than obtaining bone marrow-derived MSCs. With this background, the implantation of DFAT cells in combination with EGCG-conjugated scaffolds might be a prospective strategy to facilitate bone formation in orthopedic and maxillofacial regions. In the present study, we proposed an effective technique to promote early osteoblastic differentiation in DFAT cells using EGCG. However, further detailed research is essential to verify the *in vitro* and *in vivo* fate of the cells produced by this technique. In particular, it would be valuable to evaluate the gene expression pattern of DFAT-derived osteogenic differentiation in order to demonstrate the difference between these cells and intact osteoblasts or other osteoblastic cells. In addition, there is still no information about the main receptor of EGCG that positively and functionally promotes the osteoblast differentiation of DFAT cells. Thus, identification of such molecules is an essential step toward developing safe and reliable bone regeneration therapies using DFAT cells and EGCG.

## 4. Experimental Section

### 4.1. Chemicals

EGCG was purchased from BioVerde Inc. (Kyoto, Japan). The inhibitors of the four signaling pathways, ERK1/2 (PD98059), Akt (API-2), JNK (SP600125), and p38-MAPK (SB203580) were purchased from Sigma (Sigma-Aldrich Co. LLC, St. Louis, MO, USA).

### 4.2. Preparation and Maintenance of Primary Human DFAT Cells

DFAT cells were prepared from the subcutaneous adipose tissue of a healthy 63-year-old male patient, who underwent oral and maxillofacial surgery. This study adheres to the declaration of Helsinki, and the whole protocol was approved by the local ethics committee of Amagasaki Chuo Hospital and Osaka Dental University (approval number: 110839; 31 March 2015). Ceiling culture method was used for isolating the DFAT cells from adipose tissue as reported previously [16,24]. In brief, minced adipose tissues were filtered using a nylon mesh to eliminate undesirable components such as vascular cells, and connective tissues. Obtained mature adipocytes containing lipid droplets were then added into an overturned culture flask. The floating adipocytes adhered to the ceiling of the flask. One week later, the flask was reversed to eliminate the mature adipocytes. Thereafter, we could observe some residual mature adipocytes in the flask, and these cells were gradually removed after several passages. DFAT cells resembling fibroblastic cells occupied the bottom of the flask (Supplementary Figure S1). The DFAT cells were cultured in Dulbecco’s modified essential medium supplemented with 10% fetal bovine serum and 1% antibiotics in a 5% CO_2_ incubator at 37 °C. DFAT cells at passages 6–8 were used for each assay. Images of cell morphology were obtained using an optical microscope (IX70, Olympus, Tokyo, Japan).

### 4.3. Cell Seeding Conditions for the Proliferation Assay and Osteogenic Differentiation

To investigate the proliferation and osteoblast differentiation of the DFAT cells, the cells were seeded with the control medium under two conditions: 1 × 10^3^/well for condition 1 and 3.5 × 10^4^/well for condition 2 in 48 well plate. In condition 1, one day after the cells were seeded, they were treated with the five different media listed in Table 1. In condition 2, approximately one day after the cells were seeded, they were treated with basal medium for 17 h and then with the five different media listed in Table 1. The two conditions were applied in order to highlight the proliferation process (condition 1) and to accelerate cell differentiation (condition 2).

### 4.4. Cell Proliferation Assay

DFAT cells were seeded under condition 1. At the prescribed dates, cell proliferation was examined using the cell counting kit-8 (Dojindo Laboratories, Kumamoto, Japan) according to the manufacturer’s instruction manual.

### 4.5. RNA Isolation, Quantitative Real Time-PCR (qPCR), and Digital PCR

Total RNA was extracted using the RNeasy Mini Kit (Qiagen Inc., Valencia, CA, USA). Reverse transcription was performed with 200 ng RNA using the Transcriptor Universal cDNA Master (Roche Diagnostics, Mannheim, Germany). mRNA levels of osteogenic markers were partially analyzed by qPCR using a TaqMan Gene Expression Assay (ThermoFisher Scientific Inc., Waltham, MA, USA) on a Step One Plus PCR system (ThermoFisher Scientific Inc.). The glyceraldehyde-3-phosphate dehydrogenase (*GAPDH*) gene was used as an internal standard (Human GAPDH endogenous control; ThermoFisher Scientific Inc.). The PCR cycling conditions were as follows: 2 min at 50 °C, 20 s at 95 °C, 40 cycles of 1 s at 95 °C, 20 s at 60 °C. The signals were normalized to the *GAPDH* signal. mRNA expression levels were calculated using the comparative CT method. *RUNX2*, *OSX*, *and DLX5* expression at day 1 was evaluated using the QuantStudio 3D digital PCR system (ThermoFisher Scientific Inc.) with the QuantStudio 3D Master Mix (ThermoFisher Scientific Inc.) and Gene expression assay (ThermoFisher Scientific Inc.). The cycling conditions were as follows: 10 min at 96 °C, 39 cycles of 2 min at 60 °C, 30 s at 98 °C, and 2 min at 60 °C. The assay IDs of TaqMan Gene Expression Assay and the accession numbers used for the qPCR and digital PCR systems are as follows: *COL1A1*, Hs00164004_m1, BC036531.2; *RUNX2*, Hs01047973_m1, NM_001015051.3; *DLX5*, Hs00193291_m1, NM_005221.5; *SP7(OSX)*, Hs01866874_s1, NM_152860.1; *OCN*, Hs00609452_g1, NM_199173.4.

### 4.6. ALP Staining, and Alizarin Red Staining

Cells were seeded under condition 2 for ALP and alizarin red staining. As for ALP staining, the cells were stained at the prescribed date using the TRAP/ALP stain kit (Wako Pure Chemicals Industries Ltd., Osaka, Japan). A quantitative ALP assay was performed using LabAssay ALP (Wako Pure Chemicals Industries Ltd.). The ALP data were divided by the total quantity of DNA evaluated with Quant-iT PicoGreen ds DNA Assay Kit (ThermoFisher Scientific Inc.). All assays were performed in the accordance with manufacturer instructions.

Mineralization was assessed using alizarin red staining. In brief, the cells washed with phosphate-buffered saline were fixed with 95% ethanol for 10 min. The cultures were stained with alizarin red S (Sigma-Aldrich Co. LLC) for 30 min, followed by washing with distilled water to remove the excess dye. A Canon A495 camera (Canon, Tokyo, Japan) was used to capture images. As for the quantitative analysis of alizarin red staining, the stained wells were treated with 10% formic acid. The mixture was shaken at the room temperature for 10 min to release the dye. The absorbance of the supernatant was then evaluated with a SpectraMax M5 spectrometer (Molecular Devices, Sunnyvale, CA, USA) at 415 nm.

### 4.7. Inhibitory Assay for Four Signaling Pathways

The cell signaling pathways associated with the osteogenic differentiation of the DFAT cells induced by OM or OM(Dex) with EGCG were evaluated using four inhibitors—ERK1/2 (PD98059), Akt (API-2), p38-MAPK (SB203580), and JNK (SP600125). These inhibitors were mixed with OM + EGCG(1.25) and OM(Dex) + EGCG(1.25) at the following concentrations: 1 µM for API-2; 2.5 and 25 µM for PD98059; 1 and 10 µM for SB203580; and 1 and 10 µM for SP600125. The cells were cultured in the above-mentioned media under condition 2. At the prescribed date, the cells were stained with alizarin red S and the quantitative data of the staining were used to determine the role of each signal pathway. Relative inhibitory effect was calculated as follows: Inhibitory effect (%) = (absorbance of sample/absorbance of benchmark) × 100. Cells treated with OM or OM(Dex) with EGCG(1.25) were used as the benchmark.

### 4.8. Statistical Analysis

Statistical significance was evaluated using a student’s *t*-test and one-way analysis of variance, followed by a Tukey–Kramer test and Dunnett’s test. Microsoft Excel software statistical package was used for the calculations. All experiments were performed with at least two independent replicates and all of the results showed high reproducibility.

## 5. Conclusions

In the present study, we provided evidence indicating that supplementation of an adequate concentration of EGCG into osteogenic media is effective at inducing the proliferation, early osteogenic differentiation, and mineralization of primary human DFAT cells. OM + EGCG induced superior osteoblast differentiation in the cells than OM(Dex) + EGCG. These results indicate that supplementation of EGCG into OM might be a prospective strategy to induce the osteoblastic differentiation of DFAT cells that can be then used in bone regeneration therapy and drug screening.

## Supplementary Materials

Supplementary materials can be found at http://www.mdpi.com/1422-0067/16/12/26081/s1.
