# Supplementary material for: Application of Green Tea Catechin for Inducing the Osteogenic Differentiation of Human Dedifferentiated Fat Cells in Vitro
Source: Int J Mol Sci. 2015 Nov 25;16(12):27988–8000. doi: 10.3390/ijms161226081 (PMC4691028; doi:10.3390/ijms161226081)
Supplement: Supplementary file 1 [file ijms-16-26081-s001.pdf]

Supplementary Materials: Application of Green Tea Catechin for Inducing the Osteogenic Differentiation of Human Dedifferentiated Fat Cells *in Vitro*

Koji Kaida, Yoshitomo Honda, Yoshiya Hashimoto, Masahiro Tanaka and Shunsuke Baba

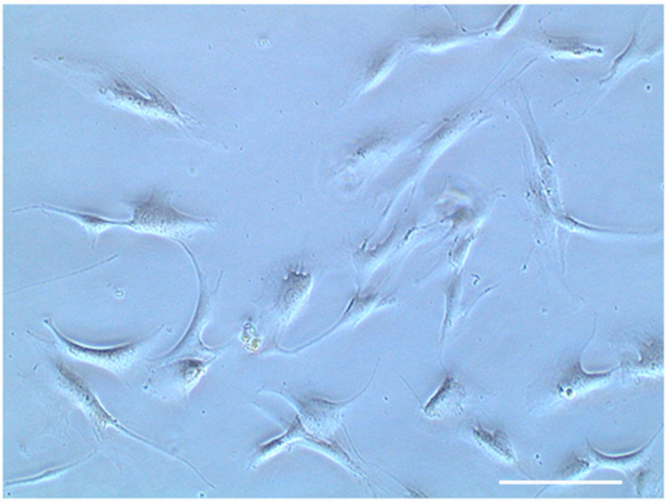

Figure S1. Representative phase contrast image of human dedifferentiated fat (DEAT) cells. Bar = 50 μm.
